# Molecular hydrogen attenuates fatty acid uptake and lipid accumulation through downregulating CD36 expression in HepG2 cells

**DOI:** 10.1186/2045-9912-3-6

**Published:** 2013-03-01

**Authors:** Akio Iio, Mikako Ito, Tomohiro Itoh, Riyako Terazawa, Yasunori Fujita, Yoshinori Nozawa, Ikuroh Ohsawa, Kinji Ohno, Masafumi Ito

**Affiliations:** 1 Department of Embryology, Institute for Developmental Research, Aichi Human Service Center, 713-8 Kamiya-Cho, Kasugai, Aichi, 480-0392, Japan; 2 Division of Neurogenetics, Center for Neurological Diseases and Cancer, Nagoya University Graduate School of Medicine, 65 Tsurumai, Showa-ku, Nagoya, Aichi, 466-8550, Japan; 3 Faculty of Agriculture, Kinki University, 3327-204 Nakamachi, Nara, Nara, 631-0052, Japan; 4 Department of Urology, Gifu University Graduate School of Medicine, 1-1 Yanagido, Gifu, Gifu, 501-1193, Japan; 5 Research Team for Mechanism of Aging, Tokyo Metropolitan Institute of Gerontology, 35-2 Sakae-cho, Itabashi, Tokyo, 173-0015, Japan; 6 Department of Food and Health, Tokai Gakuin University, 5-68 Naka-kirinocho, Kakamigahara, Gifu, 504-8511, Japan; 7 Research Team for Functional Biogerontology, Tokyo Metropolitan Institute of Gerontology, 35-2 Sakae-cho, Itabashi, Tokyo, 173-0015, Japan

**Keywords:** Molecular hydrogen, HepG2 cells, Fatty acid, JNK, Phosphorylation, CD36, Hepatic steatosis

## Abstract

**Background:**

There is accumulating evidence that obesity is closely associated with an impaired free fatty acid metabolism as well as with insulin resistance and inflammation. Excessive fatty acid uptake mediated by fatty acid translocase CD36 plays an important role in hepatic steatosis. Molecular hydrogen has been shown to attenuate oxidative stress and improve lipid, glucose and energy metabolism in patients and animal models of hepatic steatosis and atherosclerosis, but the underlying molecular mechanisms remain largely unknown.

**Methods:**

Human hepatoma HepG2 cells were exposed to palmitate-BSA complex after treatment with or without hydrogen for 24 h. The fatty acid uptake was measured by using spectrofluorometry and the lipid content was detected by Oil Red O staining. JNK phosphorylation and CD36 expression were analyzed by Western blot and real-time PCR analyses.

**Results:**

Pretreatment with hydrogen reduced fatty acid uptake and lipid accumulation after palmitate overload in HepG2 cells, which was associated with inhibition of JNK activation. Hydrogen treatment did not alter CD36 mRNA expression but reduced CD36 protein expression.

**Conclusion:**

Hydrogen inhibits fatty acid uptake and lipid accumulation through the downregulation of CD36 at the protein level in hepatic cultured cells, providing insights into the molecular mechanism underlying the hydrogen effects *in vivo* on lipid metabolism disorders.

## Background

Obesity and its associated disorders such as type 2 diabetes, coronary heart diseases and non-alcoholic fatty liver disease (NAFLD) are currently global health problems. There is accumulating evidence that obesity is closely associated with impaired free fatty acid (FFA) metabolism as well as insulin resistance and inflammation [1]. Excessive release of FFA from visceral fat adipocytes leads to the production of inflammatory and proatherogenic proteins through activation of the NFκB and c-Jun NH_2_-terminal kinase (JNK) pathways in skeletal muscle, liver and endothelial cells, and promotes atherosclerotic vascular disease (ASVD) and NAFLD.

Fatty acid translocase CD36 mediates uptake of FFA from circulation and intracellular transport of long-chain fatty acids in diverse cell types such as monocytes, platelets, macrophages, microvascular endothelial cells, adipocytes, muscle cells, enterocytes, and hepatocytes [2]. Mice deficient of CD36 exhibit defective uptake and utilization of fatty acids. Excessive fatty acid uptake mediated by CD36 plays an important role in hepatic steatosis [3]. The expression level of CD36 is very low in normal liver tissues [4], but is drastically increased in the liver tissues of high-fat diet (HFD)-induced fatty liver mice and those of human NAFLD. Conversely, forced expression of CD36 in liver causes hepatic steatosis in the absence of HFD [5]. There is extensive evidence showing that CD36 plays significant roles in hepatic steatosis, suggesting that CD36 can be a potential drug target against NAFLD.

Since the first report in 2007, which demonstrated the effect of molecular hydrogen on brain infarction [6], hydrogen has been shown to protect against a variety of diseases including oxidative stress-related diseases, inflammation and allergy in *in vivo* and *in vitro* models as well as in humans [7]. In the metabolic diseases, hydrogen attenuates oxidative stress and improves lipid, glucose and energy metabolism in patients and animal models of hepatic steatosis and atherosclerosis, but the underlying molecular mechanisms remain largely unknown [8-11]. Although the hydrogen effects have been ascribed to a selective scavenging of hydroxyl radicals, we previously reported that hydrogen attenuates type I allergy via inhibiting intracellular signaling pathways, providing the first evidence that hydrogen modulates signaling pathways [12]. We also demonstrated that hydrogen suppresses LPS/IFNγ-induced phosphorylation of apoptosis signal-regulating kinase 1 (ASK1) and its downstream signaling molecules, p38, JNK and NFκB, resulting in inhibition of iNOS expression and NO production in macrophages [13]. Based on these findings, we proposed a hypothesis that hydrogen may act as a modulator of signaling pathways, thereby exhibiting protective effects against various diseases. Consistent with our hypothesis, it has been recently reported that hydrogen inhibits signaling pathways in animal models of acute liver injury [14] and amyloid-beta-induced Alzheimer’s disease [15].

In the present study, in order to understand the underlying mechanisms of hydrogen effects on lipid metabolism disorders and atherosclerosis, we examined if hydrogen could attenuate fatty acid intake and lipid accumulation caused by palmitate overload in human hepatoma HepG2 cells. We then investigated whether hydrogen could modulate signaling pathways after palmitate overload as well as CD36 expression after hydrogen treatment in this cell culture model of hepatic steatosis.

## Materials and methods

### Cell culture and hydrogen treatment

Human hepatoma HepG2 cells were purchased from RIKEN BioResource Center (Tsukuba, Japan) and cultured in DMEM containing 10% heat-inactivated FBS in a humidified atmosphere of 5% CO_2_ at 37°C. Prior to hydrogen treatment, cells were starved in serum-free DMEM for 24 h. Hydrogen treatment was performed as described previously [12]. Briefly, cells were cultured in DMEM containing 0.67% (w/v) fatty acid-free BSA (Roche, Penzberg, Germany) under a humidified condition of 75% H_2_, 20% O_2_ and 5% CO_2_, or 95% air and 5% CO_2_ in a small aluminum bag. After treatment with or without hydrogen for 24 h, cells were treated with 0.67% fatty acid-free BSA or with 0.3 and 1.0 mM sodium palmitate (Sigma, St. Louis, MO, USA)-BSA complex (containing 0.67% fatty acid-free BSA) for 24 h to analyze the lipid content. Cells were also treated with fatty acid-free BSA or with 0.3 mM sodium palmitate-BSA complex for 120 min to analyze the protein phosphorylation.

### Cell viability assay

After treatment with or without hydrogen for 24 h, cell viability was determined calorimetrically using the Cell Counting kit (WST-1 assay: Wako, Osaka, Japan) according to the manufacturer’s protocol.

### Measurement of fatty acid uptake and lipid content

Fatty acid uptake assay was performed as described by Liao et al. [16] with slight modification. After treatment with or without hydrogen for 24 h, cells were washed twice with Hank’s balanced salt solution (HBSS: Gibco, Langley, OK, USA) and incubated in HBSS containing 0.1% fatty acid-free BSA and 0.5 μg/ml BODIPY FL C16 (Molecular Probes, Eugene, OR, USA) for 15 min at 37°C. After washing twice with ice-cold HBSS containing 0.2% BSA, cells were detached with 10 mM EDTA/PBS and subjected to the measurement of fluorescence using the MT-600 F fluorescence microplate reader (Corona Electric, Hitachinaka, Japan). The relative BODIPY FL C16 uptake was expressed as fluorescence intensity in cells relative to the total amount of protein. To quantify the lipid content, cells were stained with Oil Red O for 10 min and then dye was extracted and measured as described previously [17].

### CT-B binding assay

After treatment with or without hydrogen for 24 h, cells were washed twice with HBSS and incubated in HBSS containing 0.1% fatty acid-free BSA and 0.5 μg/ml Alexa594-conjugated cholera toxin B subunit (CT-B; Molecular Probes) for 1 h at 37°C. After washing twice with ice-cold HBSS containing 0.2% BSA, cells were subjected to the measurement of fluorescence using the fluorescence microplate reader.

### Real-time RT-PCR analysis

Total RNA was extracted from cells by Isogen II (Wako) followed by DNase I treatment. cDNA was synthesized using the PrimeScript RT reagent kit (Takara, Ohtsu, Japan) and quantitative real-time PCR was performed using SYBR Premix Ex Taq II (Tli RNaseH Plus: Takara) and the real-time thermal cycler Dice (Takara). Primer sets were as follows: GAPDH, 5’-CCACATCGCTCAGACACCAT-3’ and 5’-GCAACAATATCCACTTTACCAGAGTTAA -3’ [18]; CD36, 5’-TGGAACAGAGGCTGACAACTT-3’ and 5’-TTGATTTTGATAGATATGGGATGC-3’. The expression level of *CD36* gene was determined using the comparative C_t_ method and normalized to that of *GAPDH*.

### Western blot analysis

Whole cell extracts were prepared by using RIPA buffer containing the protein inhibitor cocktail (Roche) and the phosphatase inhibitor cocktail (Sigma). Samples were subjected to sodium dodecyl sulfate-polyacrylamide gel electrophoresis (SDS-PAGE) and electroblotted onto PVDF membranes. Membranes were incubated with a primary antibody, anti-SAPK/JNK, anti-phospho-SAPK/JNK (Thr183/Tyr185), anti-GAPDH (Cell Signaling Technology, Beverly, MA, USA) or anti-CD36 (GenTex, Irvine, CA, USA), followed by incubation with a horseradish peroxidase-conjugated anti-rabbit secondary antibody. Protein bands were detected using the ECL Plus (GE Healthcare, Little Chalfont, UK) and the chemiluminescence imager LAS 4000 (Fujifilm, Tokyo, Japan).

### Statistical analysis

Results were expressed as mean ± SD of three independent experiments unless otherwise noted. Data were analyzed using Student’s *t*-test. A value of *p* < 0.05 was considered significant.

## Results and discussion

### Hydrogen does not affect viability of HepG2 cells

**Figure 1:**
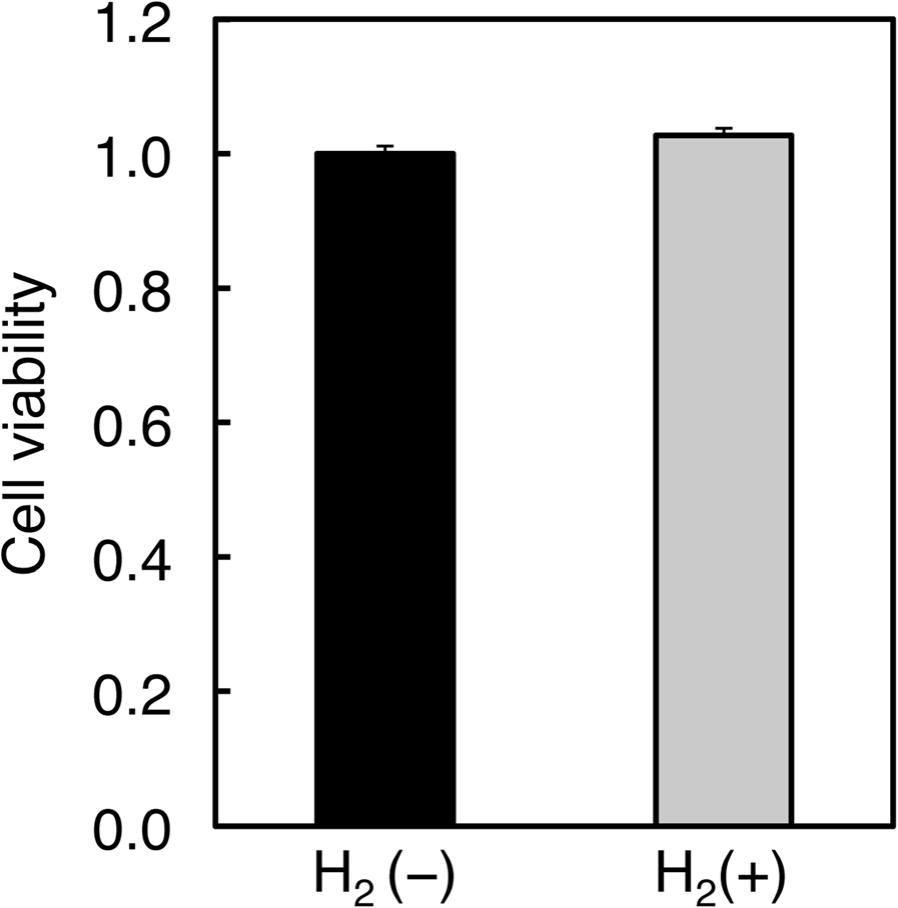
**Effects of hydrogen on viability of HepG2 cells.** After serum starvation, HepG2 cells were treated with or without hydrogen for 24 h and cell viability was determined by the WST-1 assay.

We determined the effects of hydrogen on viability and morphology of HepG2 cells under the experimental condition we described previously [12]. After serum deprivation for 24 h, cells were treated with hydrogen in DMEM containing fatty acid-free BSA for 24 h and then subjected to cell viability assay. Hydrogen treatment did not affect cell viability (Figure 1) and morphology (data not shown). This is consistent with our previous finding that hydrogen did not influence cell viability and morphology of cultured cells when administered according to our protocol [12,13]. These results suggest that hydrogen is unlikely to induce cell death or proliferation in subsequent experiments.

### Hydrogen reduces fatty acid uptake and lipid accumulation

**Figure 2:**
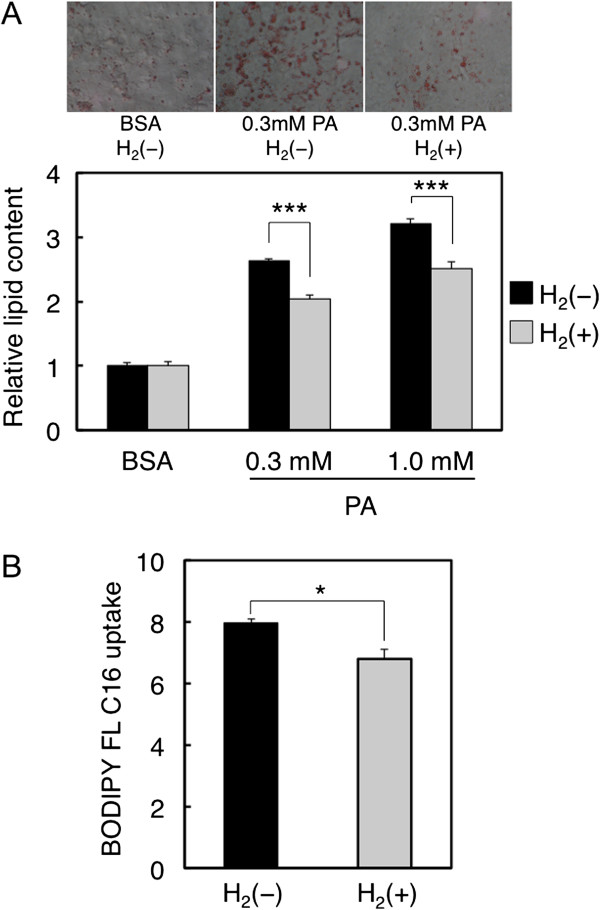
**Effects of hydrogen on fatty acid uptake and lipid accumulation in HepG2 cells.** (**A**) After treatment with or without hydrogen for 24 h, cells were exposed to 0.3 mM palmitate-BSA (PA) for 24 h, and then stained with Oil Red O. The Oil Red O content was measured at a wavelength of 540 nm. Data are expressed as mean ± SD (n = 6) (***p < 0.001). (**B**) After treatment with or without hydrogen for 24 h, cells were exposed to BODIPY-FL C16 for 15 min and fluorescence intensity was measured (*p < 0.05).

In patients with type 2 diabetes, oral intake of hydrogen-rich water is associated with the reduction of serum concentrations of oxidized low-density lipoprotein (LDL) and FFA [9], both of which are CD36 ligands. Administration of hydrogen-rich water prevents atherosclerosis in apolipoprotein E knockout mice, a model of the spontaneous development of atherosclerosis [19]. However, the molecular mechanisms of hydrogen to improve lipid metabolism and atherosclerosis remain largely unknown. In an attempt to investigate the underlying mechanisms, we first examined whether hydrogen could exhibit beneficial effects on lipid metabolism in a cell culture model of hepatic steatosis. According to a previous report by Gómez-Lechón et al. [20], an overload of fatty acids leads to lipid accumulation in HepG2 cells. We thus treated HepG2 cells with 0.3 or 1.0 mM palmitate-BSA for 24 h and then performed Oil Red O staining to detect the intracellular triglyceride (TG) content. Indeed, incubation of the cells with palmitate-BSA developed a clear increase of lipid accumulation in the cytosol compared with the BSA control (Figure 2A). We then studied if pretreatment with hydrogen for 24 h could affect lipid accumulation by palmitate overload. As shown in Figure 2A, intracellular lipid accumulation caused by 0.3 or 1.0 mM palmitate was significantly reduced by hydrogen treatment. In apolipoprotein E knockout mice, atherosclerotic lesions were decreased by hydrogen as judged by Oil Red O staining of macrophages in serial sections of the aorta [19]. Our results and others suggest that molecular hydrogen is capable of reducing fat accumulation in *in vitro* and *in vivo* models of metabolic diseases.

In hepatocytes, FFA is converted to TG, which is used for production of very low-density lipoprotein (VLDL), a class of lipoproteins. VLDL transports TG from the liver and intestine to adipose and muscle tissues, but excess TG is stored in lipid droplets [3]. The cellular lipid content in part depends on FFA uptake through transmembrane transport. In order to examine if hydrogen could influence FFA uptake, cells were treated with a BODIPY-labeled fluorescent fatty acid analog, BODIPY FL C16, for 15 min after treatment with or without hydrogen for 24 h. After washing, intracellular fluorescence was measured. As shown in Figure 2B, the fluorescent-labeled palmitate uptake was significantly reduced by hydrogen treatment. Taken together, these results suggest that hydrogen inhibits fatty acid uptake and lipid accumulation after palmitate overload in HepG2 cells.

### Hydrogen inhibits palmitate-induced phosphorylation of JNK

**Figure 3:**
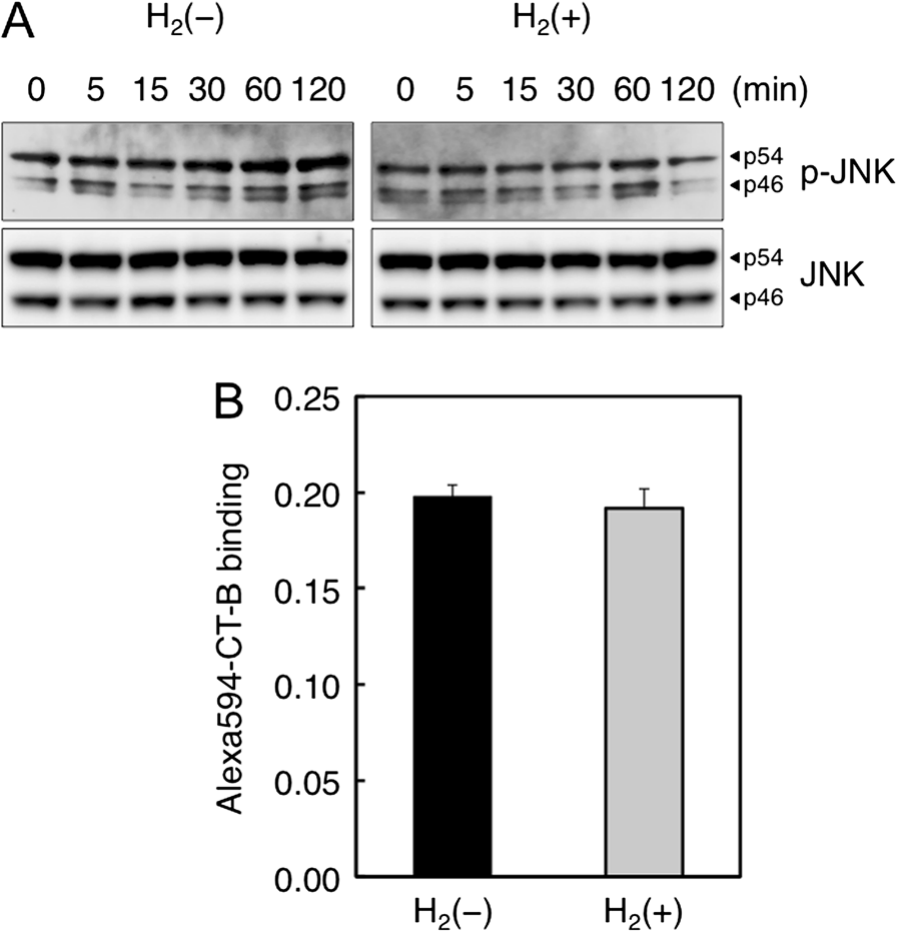
**Effects of hydrogen on JNK phosphorylation and lipid raft integrity in HepG2 cells.** (**A**) After treatment with or without hydrogen for 24 h, cells were incubated in the presence of palmitate-BSA (0.3 mM) for indicated time periods, and cell lysates were harvested and subjected to Western blot analysis for JNK and phospho-JNK proteins. (**B**) After treatment with or without hydrogen for 24 h, HepG2 cells were exposed to Alexa594-CT-B for 1 h at 37°C and fluorescent intensity was measured with the fluorescence microplate reader.

In an attempt to elucidate molecular mechanisms underlying the reduction by hydrogen of fatty acid uptake and lipid accumulation in HepG2 cells, we investigated the effect of hydrogen on palmitate-induced activation of signaling pathways. Since Gao et al. have recently reported that FFA induces hepatic insulin resistance through JNK and p38 MAPK (mitogen-activated protein kinases) pathways [21], we analyzed JNK and p38 activation after palmitate treatment in HepG2 cells. As shown in Figure 3A, phosphorylation of JNK caused by palmitate overload was attenuated by hydrogen treatment. In contrast, activation of p38 was not affected by hydrogen (data not shown). Among the physiological responses induced by fatty acid overload, JNK activation has been shown to be the most responsible for palmitate-induced oxidative stress and HFD-induced hepatic steatosis [22,23]. Hence, our results suggest that inhibition of JNK activation by hydrogen may contribute to the attenuation of palmitate-induced hyperlipogenesis.

### Hydrogen downregulates protein expression of CD36

Binding of CT-B to ganglioside GM1 is a marker to identify lipid rafts, which are membrane microdomains enriched in cholesterol and sphingolipid [24]. There are several evidences for the correlation of the level of FFA uptake with the expression level of FFA transporter proteins and with the integrity of lipid rafts [25]. In order to examine if hydrogen affected the lipid raft integrity in HepG2 cells, we measured the intensity of fluorescent-labeled CT-B binding after hydrogen treatment using a fluorescence microplate reader. As shown in Figure 3B, hydrogen treatment did not affect the lipid raft integrity. These findings suggested the possibility that inhibition of FFA uptake by hydrogen might be due to the altered expression of FFA transporter proteins such as CD36.

**Figure 4:**
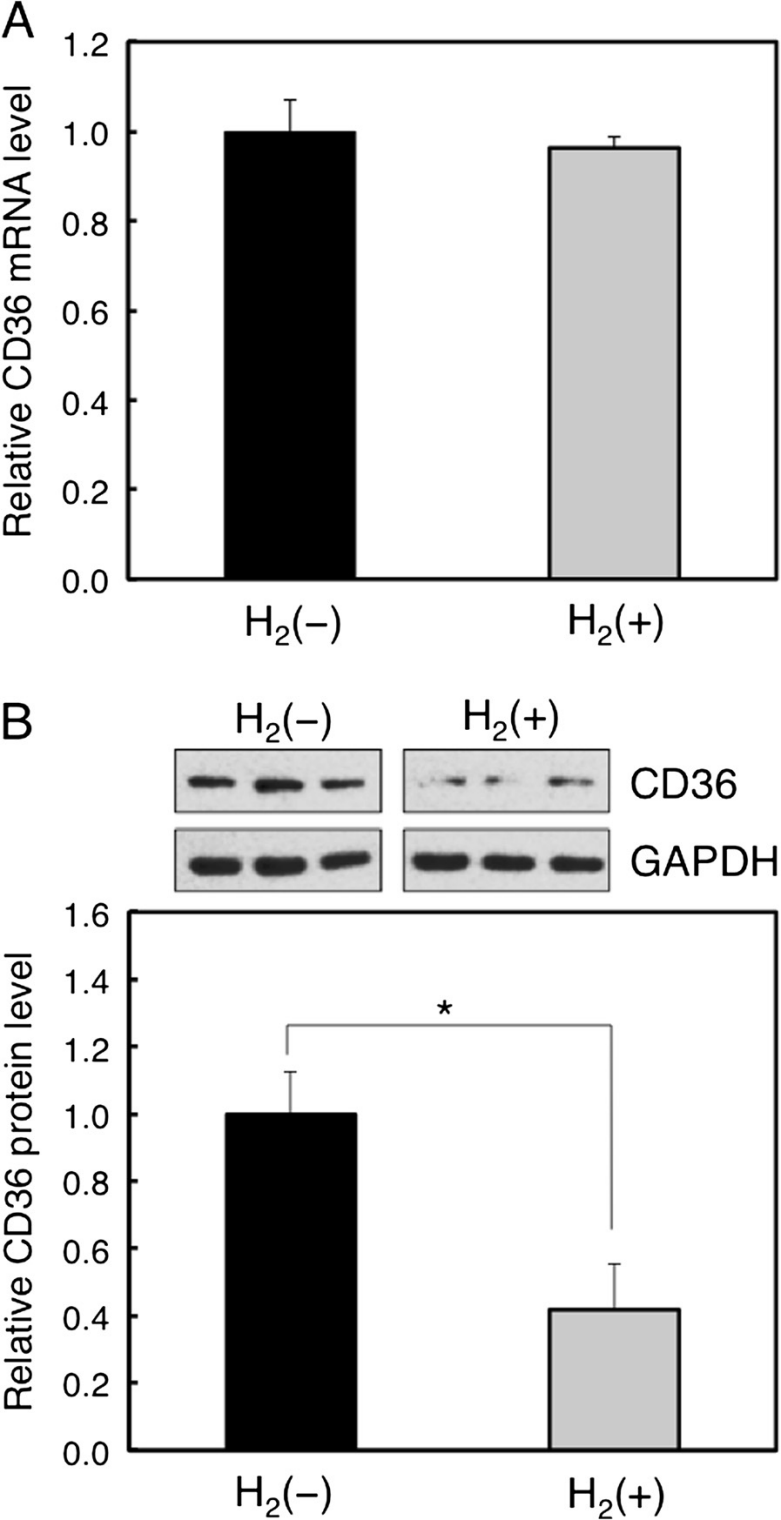
**Effects of hydrogen on CD36 expression in HepG2 cells.** After treatment with or without hydrogen for 24 h, total RNA was harvested and quantitative real-time RT-PCR was performed for CD36 mRNA (**A**), and cell lysates were harvested and Western blot analysis was performed for CD36 protein (**B**). GAPDH was used as an internal control, and the CD36 protein level (fold) was evaluated by densitometry (*p < 0.05).

To verify whether hydrogen attenuated palmitate-induced lipid accumulation in HepG2 cells through regulation of CD36 expression, the expression level of CD36 after hydrogen treatment was evaluated by real-time PCR and Western blot analyses. Unexpectedly, CD36 mRNA expression was not altered by hydrogen treatment (Figure 4A), but CD36 protein expression was significantly downregulated by hydrogen (Figure 4B). It has been previously reported that a non-physiological short chain ceramide, C_2_-ceramide, reduces CD36 expression at protein levels but not at mRNA levels in monocytes and macrophages [26]. Furthermore, the antiretroviral protease inhibitor, ritonavir, which inhibits proteasome activity in THP-1 cells, increases CD36 protein levels but not mRNA levels, followed by increased oxLDL uptake and cholesterol levels [27]. These findings suggest that the reduction of CD36 protein expression by hydrogen treatment may involve post-transcriptional events including inhibition of protein translation or enhancement of protein degradation. Ubiquitination plays an important role in acute regulation of many membrane protein levels. Smith et al. found that CD36 protein was ubiquitinated on lysines 469 and 472 on its C-terminal domain [28]. This process is enhanced by fatty acid, indicating the negative feedback mechanism of CD36 expression. It would be possible that hydrogen might activate the ubiquitin-proteasome system and downregulate CD36 expression. A recent report also showed that Parkin mono-ubiquitinates CD36, stabilizing and increasing CD36 [29]. If hydrogen could inhibit the activity or expression of Parkin, CD36 might be poly-ubiquitinated and rapidly degraded, the possibility of which remains to be studied.

### Hydrogen downregulates CD36 protein expression and thereby inhibits palmitate-induced phosphorylation of JNK

In the present study, hydrogen treatment results in downregulation of CD36 protein expression in HepG2 cells, which inhibits fatty acid uptake when cells are subjected to palmitate overload. Therefore, inhibition of palmitate-induced phosphorylation of JNK is likely to be the consequence of the reduced uptake of fatty acid. Our results suggest that downregulation of CD36 expression by hydrogen pretreatment may be the primary mechanism against hepatic steatosis in this in vitro model.

Finally, it is worth to note that we investigated the molecular mechanisms of the hydrogen effects on lipid metabolism using the cell culture system we previously developed. To keep the hydrogen concentration high in the medium, cells are cultured under the condition of 75% H_2_, 20% O_2_, and 5% CO_2_. A recent report, however, demonstrated that oral intake of water containing a relatively lower concentration of hydrogen was effective in an animal model of Parkinson’s disease [30]. Furthermore, the increase in hydrogen concentration in the body after taking hydrogen water should be transient. In order to precisely recapitulate the hydrogen effects in vivo, a novel cell culture system needs to be developed in which concentration and timing of hydrogen treatment can be readily changed.

## Conclusions

Hydrogen downregulates the protein expression of CD36, and inhibits fatty acid uptake and lipid accumulation in HepG2 cells. As the consequence, hydrogen may modulate signal transduction such as the JNK pathway. Our results provide insights into the molecular mechanism underlying the hydrogen effects on lipid metabolism disorders such as hepatic steatosis and NAFLD.

## Abbreviations

NAFLD: Non-alcoholic fatty liver disease; FFA: Free fatty acid; JNK: c-Jun NH_2_-terminal kinase; ASVD: Atherosclerotic vascular disease; HFD: High-fat diet; ASK1: Apoptosis signal-regulating kinase 1; HBSS: Hank’s balanced salt solution; CT-B: Cholera toxin B subunit; SDS-PAGE: Sodium dodecyl sulfate-polyacrylamide gel electrophoresis; LDL: Low-density lipoprotein; TG: Triglyceride; VLDL: Very low-density lipoprotein; MAPK: Mitogen-activated protein kinases

## Competing interests

The authors declare that they have no competing interests.

## Authors’ contributions

IO, KO, MI and YN participated in the design of the study. MiI and TI set up the system of hydrogen treatment in *in vitro* culture system. AI, MiI, RT and YF carried out experiments. All authors read and approved the final manuscript.
